# Benzene degradation in a denitrifying biofilm reactor: activity and microbial community composition

**DOI:** 10.1007/s00253-017-8214-8

**Published:** 2017-03-20

**Authors:** Marcelle J. van der Waals, Siavash Atashgahi, Ulisses Nunes da Rocha, Bas M. van der Zaan, Hauke Smidt, Jan Gerritse

**Affiliations:** 1 Deltares, Subsurface and Groundwater Systems, Princetonlaan 6, 3584 CB Utrecht, The Netherlands; 2 Wageningen University & Research, Laboratory of Microbiology, Stippeneng 4, 6708 WE Wageningen, The Netherlands; 3 VU University of Amsterdam, Department of Molecular Cell Physiology, De Boelelaan 1085, 1081 HV Amsterdam, The Netherlands

**Keywords:** Anaerobic benzene degradation, Denitrification, *Peptococcaceae*, Biofilm reactor, Retentostat, Carboxylation

## Abstract

Benzene is an aromatic compound and harmful for the environment. Biodegradation of benzene can reduce the toxicological risk after accidental or controlled release of this chemical in the environment. In this study, we further characterized an anaerobic continuous biofilm culture grown for more than 14 years on benzene with nitrate as electron acceptor. We determined steady state degradation rates, microbial community composition dynamics in the biofilm, and the initial anaerobic benzene degradation reactions. Benzene was degraded at a rate of 0.15 μmol/mg protein/day and a first-order rate constant of 3.04/day which was fourfold higher than rates reported previously. Bacteria belonging to the *Peptococcaceae* were found to play an important role in this anaerobic benzene-degrading biofilm culture, but also members of the *Anaerolineaceae* were predicted to be involved in benzene degradation or benzene metabolite degradation based on Illumina MiSeq analysis of 16S ribosomal RNA genes. Biomass retention in the reactor using a filtration finger resulted in reduction of benzene degradation capacity. Detection of the benzene carboxylase encoding gene, *abcA*, and benzoic acid in the culture vessel indicated that benzene degradation proceeds through an initial carboxylation step.

**Electronic supplementary material:**

The online version of this article (doi:10.1007/s00253-017-8214-8) contains supplementary material, which is available to authorized users.

## Introduction

Benzene is a constituent of crude oil and gasoline and is often considered as risk-determining compound at contaminated locations, because it easily dissolves and transports in groundwater and is carcinogenic (Chen et al. 2012). Benzene may enter the groundwater from for example leaking storage tanks or pipelines. Benzene is readily biodegradable under aerobic conditions (Suarez and Rifai 1999). However, once contaminants such as benzene reach the groundwater, oxygen is usually depleted by microbial respiration (Lovley 1997). Consequently, anaerobic degradation is essential for benzene removal from such oxygen-depleted subsurface environments. The biggest challenge for anaerobic benzene biodegradation is to overcome the compound’s extreme chemical stability in the absence of molecular oxygen. Three mechanisms for the initial step of anaerobic benzene degradation have been proposed: (1) benzene hydroxylation, (2) benzene carboxylation, and (3) benzene methylation, leading to the formation of respectively phenol, benzoate, and toluene as the main metabolites (Meckenstock et al. 2016; Vogt et al. 2011; Weelink 2008).

Benzene degradation under nitrate-reducing conditions has been demonstrated in enrichments (Burland and Edwards 1999; Dou et al. 2008a, b; Kasai et al. 2006; Nales et al. 1998; Ulrich and Edwards 2003; van der Zaan et al. 2012) and pure cultures (Dou et al. 2010). *Dechloromonas* strains RCB and JJ (Coates et al. 2001), two *Azoarcus* strains (DN11 and AN9) (Kasai et al. 2006), and *Bacillus cereus* (Dou et al. 2010) have been isolated for their ability to degrade benzene with nitrate as electron acceptor. The genome of *Dechloromonas* strain RCB does not include the genes encoding for benzylsuccinate synthase or enzymes involved in the benzoyl-CoA pathway for monoaromatics (Salinero et al. 2009). The isolated strains might therefore degrade benzene through intra-aerobic pathways, i.e., using oxygen released from nitrogen reduction. Hence, to the best of our knowledge, there has been no report on the isolation of strictly anaerobic denitrifying benzene degraders. Recently, bacteria related to the anaerobic gram-positive *Peptococcaceae* have been shown to play an important role in anaerobic benzene degradation under denitrifying (Luo et al. 2014; van der Zaan et al. 2012), iron-reducing (Abu Laban et al. 2010; Kunapuli et al. 2007), and sulfate-reducing conditions (Abu Laban et al. 2009; Herrmann et al. 2010; Kleinsteuber et al. 2008; Taubert et al. 2012; van der Zaan et al. 2012).

Microbial community dynamics can be efficiently studied using a combination of generic high-throughput analysis of 16S ribosomal RNA (rRNA) genes through next generation technology sequencing (e.g., Illumina MiSeq) of PCR-barcoded amplicons, and quantitative-PCR (qPCR) targeting 16S rRNA genes of specific microbial taxa or genes involved in key catabolic reactions (e.g., benzene degradation). However, only a limited number of qPCR assays targeting key catabolic genes are currently available. Available functional gene qPCR assays for the degradation of benzene and potential main metabolites target benzylsuccinate synthase α-subunit (*bssA*) involved in anaerobic toluene degradation (Winderl et al. 2008), and 6-oxocyclohex-1-ene-1-carbonyl-coA hydrolase (*bamA*) (Ruan et al. 2016), involved in the reductive benzene ring cleavage. A qPCR assay targeting benzene carboxylase genes that could give information on benzene degradation through the proposed carboxylation pathway is currently not available (Lueders 2017).

We studied an anaerobic benzene-degrading microbial consortium, which has been enriched in a continuous culture with nitrate as electron acceptor for more than 14 years. Over these years, microbial biofilms have formed on the glass and metal surfaces within the reactor vessel. Besides nitrate, this consortium is able to couple benzene degradation to reduction of chlorate, ferric iron, or sulfate as electron acceptors (van der Zaan et al. 2012). From previous studies, it is known that after many years of enrichment, still a diverse microbial community is present in the continuous culture vessel (van der Zaan et al. 2012). DNA-stable isotope probing with ^13^C-labeled benzene indicated that members of the *Peptococcaceae* are dominant benzene degraders presumably involved in the initial benzene ring activation. Recently, bacteria belonging to the *Ochrobactrum* sp. and *Bacillus* sp. possibly capable of anaerobic benzene degradation with nitrate as electron acceptor were isolated from the same continuous culture (Balk, personal communication). For effective benzene bioremediation practices in the field, detailed studies on the degradation capabilities and mechanisms of the anaerobic benzene-degrading organisms are necessary. To that end, the objectives of this study were: (i) to determine the maximum benzene degradation capacity of this consortium under a variety of controlled continuous culture conditions, (ii) to obtain a highly active benzene-degrading community, (iii) to characterize differences in the microbial composition in the biofilm and liquid phase of the reactor and activity of the microbial populations involved, and (iv) to provide further mechanistic insight in the initial step in benzene degradation.

## Materials and methods

The anaerobic benzene-degrading microbial community is found predominantly attached to the glass and metal surfaces of the culture vessel submerged in the growth medium. Originally, the continuous culture was inoculated with soil from a benzene-contaminated site located in the northern part of the Netherlands (van der Zaan et al. 2012). The experiments were done with the microbial community in the continuous culture before and after the addition of a filtration finger in an attempt to further increase the activity of the benzene-degrading culture. Continuous culture experiments were performed to obtain the first-order benzene degradation rate constant, *k*, and the specific benzene degradation rate, *r*_max_, of the microbial community. The composition of the communities residing in the reactor was studied by molecular techniques. LC-qTOF-MS measurements and qPCR were used to reveal the anaerobic benzene degradation mechanism.

### Continuous culture

The anaerobic continuous culture was operated for more than 14 years on benzene as the sole electron donor under nitrate-reducing conditions as described previously (van der Zaan et al. 2012). The system was adapted for this study as follows: (i) the influent medium contained per liter: 0.5 g KH_2_PO_4_, 0.25 g (NH_4_)_2_SO_4_, 0.4 g NaNO_3_, 10 ml trace element solution SL-4 (DSMZ medium 462, www.dsmz.de), and 1 ml trace element solution SL+ (contained per liter: 0.5 g NaOH, 3 mg Na_2_SeO_3_, 4 mg Na_2_WO_4_); (ii) the pH of the medium was 6.7; (iii) the temperature in the culture vessel was maintained at 25 °C and the liquid phase was stirred at 200 rpm; (iv) the headspace was flushed with N_2_/CO_2_ (80%/20%) and the gas flowrate was kept proportional (2.4-fold higher) to the medium pump rate using a Marprene pump tube (Watson-Marlow, MA, USA). The medium was kept under a N_2_/CO_2_ (80%/20%) atmosphere and added via viton tubing (Rubber BV, Hilversum, The Netherlands) and a PVC pump tube (Watson-Marlow). Benzene was injected from a 22 mM anaerobic aqueous stock solution directly into the liquid phase using a syringe pump (KDS-230-CE, kdScientific, MA, USA) and viton tubing (Rubber BV). The mixed N_2_/CO_2_ gas was passed over a 460 ml glass column filled with hot copper filings (250 °C) to remove traces of oxygen before entering the growth vessel. The benzene degradation rate constant was obtained by changing the dilution rate, i.e., by changing the pump rates of the medium and benzene proportionally. The specific benzene degradation rate was calculated using the total protein content in the culture vessel in both the liquid and biofilm. To determine whether benzene degradation was diffusion limited in the biofilm in the culture vessel, the stirring rate of the reactor was varied between 100 and 200 rpm.

Transfers of liquid with aggregates of biofilm from the continuous culture to microcosms were supplied with 100 μM benzene and 3.6 mM nitrate to obtain microcosm enrichments. To test growth on benzoate, 10% (*v*/*v*) liquid from the reactor was transferred to microcosms with 10 mM benzoate and 40 mM nitrate.

### Retentostat experiment

**Fig. 1:**
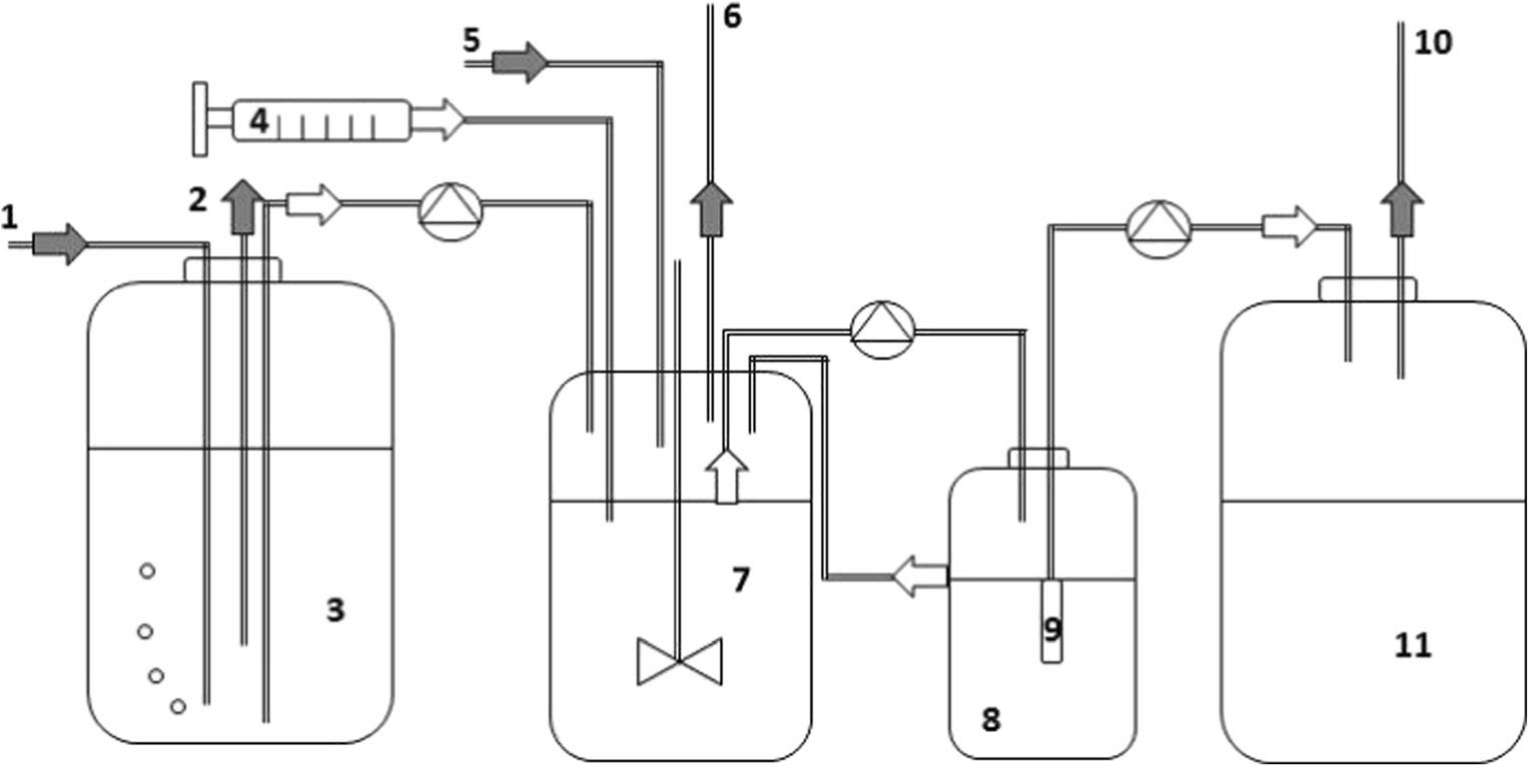
Schematic presentation of experimental set up of the reactor vessel with retentostat finger. The medium (3) kept under N_2_/CO_2_ (*1* and *2*) and benzene (*4*) were continuously added to the N_2_/CO_2_ flushed (*5* and *6*) continuous culture (*7*). The recirculation batch (*8*) with retentostat finger (*9*) was used to filter (0.22 μm pore size) the liquid phase pumped to the effluent tank (*11*). Gas is released from the effluent tank directly to the atmosphere (*10*). Due to the pressure difference liquid is flowing back from the recirculation batch to the continuous culture (7)

A retentostat system with a filtration finger was used in an attempt to further increase the activity of the benzene-degrading culture by retaining biomass in the reactor vessel. To this end, a bottle with filtration finger (Ercan et al. 2015; Van Verseveld et al. 1986) (VU University, Molecular Cell Physiology, Amsterdam, The Netherlands, cellulose acetate filter; 0.22 μm, Millipore, MA, USA) was added to the system (Fig. 1). Liquid was recirculated between the reactor vessel and the bottle with the filtration finger. Approximately 10% of the recirculating liquid was pumped through the finger to the effluent vessel.

### Analyses

Benzene was measured with a Varian 3800 gas chromatographic (GC) system equipped with a flame ionization detector (FID), a CP-PoraBond Q column (0.32 mm × 25 m), and a retention gap (methyl deactivated, 0.32 mm x 2 m) (Varian, Middelburg, The Netherlands). The FID detector was set at 300 °C, and the sample was injected at 200 °C with a split ratio of 5:1. The oven temperature program was 3 min at 50 °C, followed by an increase of 10 °C/min to 250 °C for 2 min. The flow rate of helium carrier gas was 2 ml/min. Continuous culture headspace samples of 1 ml were taken with a 1-ml Pressure-Lock gas syringe (Alltech, Breda, The Netherlands) followed by 0.5 ml injection into the GC-FID. The benzene concentration in the liquid is expressed in micromolar (µM) assuming complete dissolution of benzene. For calibration six standards ranging from 10 to 200 μM, benzene was prepared in serum bottles with the same headspace/water ratio (*v*/*v*) as in the continuous culture. The bottles were crimp sealed with viton stoppers and aluminum caps. The liquid and headspace were flushed with N_2_/CO_2_ (80%/20%). The coefficient of variation of this GC method was 5%, and the benzene detection limit was 0.1 μM. Benzoate was measured using a LC-qTOF-MS as performed previously (Helmus et al. 2016). Liquid samples were conserved with 25 mg/l mercury chloride before LC-qTOF-MS analysis.

Continuous culture biofilm and liquid samples were analyzed for protein content at a dilution rate of 2/day, according to the procedure of Lowry et al. (1951). Six standards ranging from 0 to 500 μg protein/ml of bovine serum albumin (BSA) were used for calibration. The total amount of biofilm protein in the culture vessel was assessed by extrapolating the amount of protein released from defined surface areas as described below to the total area of biofilm covered surfaces in the system.

### Biofilm sampling

Continuous culture samples for analyses of changes in microbial composition and activity (nucleic acids and protein) were taken by scraping off defined areas of biofilm attached to the glass wall under a constant N_2_/CO_2_ (80%/20%) flow. Subsequently, the liquid phase in the vessel was stirred for 5 min at 200 rpm to disperse the biofilm aggregates. Samples containing biofilm aggregates were taken directly from the liquid phase of the culture vessel using a 60 ml syringe via a sampling pipe and viton tubing (Rubber BV). The samples were immediately stored at −80 °C until DNA extraction. Different areas of biofilm with different morphology, respectively white and brown biofilm, were sampled (Fig. S1). Sample_1 contained 6.25 cm^2^ white biofilm (11% biofilm and 89% liquid protein). Sample_2 contained also 6.25 cm^2^ white biofilm, corresponding to 11% biofilm and 89% liquid protein (sample taken 3 days later compared with Sample_1). Samples 3 and 4 contained 22.25 cm^2^ white and 4 cm^2^ brown biofilm corresponding to 70% biofilm and 30% liquid protein and 22.25 cm^2^ white and 8 cm^2^ brown biofilm corresponding to 100% biofilm, respectively.

### DNA extraction, 16S rRNA gene amplicon sequencing and data analysis

Total DNA was extracted from the biofilm samples using a CTAB/phenol-chloroform method as described previously (Rajeev et al. 2013). Illumina MiSeq amplicon sequencing of the extracted DNA was used for bacterial community analysis. Barcoded amplicons from the V1-V2 region of 16S rRNA genes were generated using a two-step PCR method as described earlier (Atashgahi et al. 2016). In brief, the first amplification of partial bacterial 16S rRNA genes was done using primer pair 27F-DegS and 338R-I+II in 50 μl PCR reactions. The primers were 5′- UniTag-extended (Tian et al. 2016). The second amplification was done using 85 μl mastermix, 10 μl barcoded primers targeting the UniTag extensions, and 5 μl PCR product from the first PCR. Afterwards, barcoded PCR products were cleaned using the HighPrep PCR clean-up system (Magbio Genomics, MD, USA), quantified using Qubit (Thermo Fisher Scientific, MA, USA), and pooled in equimolar amounts as previously described (Atashgahi et al. 2016). Samples were sequenced by GATC (GATC Biotech AG, Constance, Germany) on a MiSeq platform. 16S rRNA amplicon sequence analysis was done using NG-Tax (Ramiro-Garcia et al. 2016). Briefly, paired-end libraries were filtered to retain read pairs with perfectly matching barcodes. 16S rRNA gene sequences were clustered into operational taxonomic units (OTUs) at >98.5% sequence similarity. OTUs were assigned using an open reference approach and a customized SILVA 16S rRNA database version 111 (Quast et al. 2013). The phylogenetic diversity (PD whole tree) was calculated using Quantitative Insights Into Microbial Ecology (QIIME) (Caporaso et al. 2010). Quality filtering of the sequences was done using defined ‘Mock’ communities as a positive control to deal with issues associated with filtering parameter optimization (Ramiro-Garcia et al. 2016). Two different Mock communities used in this analysis had a Pearson correlation at genus level of 0.73 and 0.78, respectively, with respect to the microbial community theoretically present in the Mock community, which are values routinely observed in other runs using the same set-up. Nucleotide sequence data reported are available at the European Nucleotide Archive (ENA) under accession number PRJEB18709.

### Quantification of total bacterial 16S rRNA genes, *bamA*, and *bssA* genes

An overview of the used primer pairs, probes, and thermal cycling conditions for total bacterial 16S rRNA, *bssA*, and *bamA* genes is shown in Table 1. Amplification of total bacterial 16S rRNA genes was carried out in a 25 μl reaction mixture containing 0.6 μM of each primer, 3 μl template DNA, 6.5 μl MilliQ, and 12.5 μl 2x IQ SYBR Green Supermix (Bio-Rad). The lower detection limit of the assay was 53.7 gene copies/μl sample defined at the amount of cycles at the lowest detected calibration sample plus 1 cycle. Amplification of *bamA* genes was performed in a 25 μl PCR reaction mixture as described for the total bacterial 16S rRNA gene assay with the addition of 0.4 μg/μl BSA. Both assays were performed on a CFX96 real-time PCR machine (Bio-Rad). The lower detection limit was 6.3 gene copies/μl sample. Amplification of *bssA* genes was done with the addition of a Taqman FAM probe (Beller et al. 2002). The lower detection limit of the *bssA* gene assay was 3.1 gene copies/μl sample. PCR amplification was carried out on an IQ5 real-time PCR system (Bio-Rad) using 0.6 μM of each primer, 3 μl template DNA, 6.3 μl MilliQ, 12.5 μl 2x IQ Supermix (Bio-Rad), and 0.08 μM probe (Eurofins MWG Operon, Ebersberg, Germany).

**Table 1:** qPCR primers and thermal cycling conditions used in this study

Target	Oligonucleotide sequence (5′-3′)^a, b^	Thermal profile	Number of cycles	References
16S rRNA gene total bacteria	GCCAGCAGCCGCGGTAAT (519F) CCGTCAATTCCTTTGAGTTT (907R)	94 °C 3 min 94 °C 30 s, 58 °C 30 s, 72 °C 30 s 72 °C 5 min Melt curve from 58 °C to 95 °C with increment 0.5 °C/10 s	1 35 1 1	519F (Lane 1991) 907R (Muyzer and Ramsing 1995)
*bamA*	GCAGTACAAYTCCTACACSACYGABATGGT (oah_F) CCRTGCTTSGGRCCVGCCTGVCCGAA (oah_R)	95 °C 5 min 95 °C 15 s, 58 °C 30 s, 72 °C 30 s 72 °C 10 min Melt curve from 58 °C to 95 °C with increment 0.5 °C/10 s	1 50 1 1	(Staats et al. 2011)
*bssA*	ACGACGGYGGCATTTCTC (bssA_F) GCATGATSGGYACCGACA (bssA_R) FAM CTTCTGGTTCTTCTGCACCTTGGACACC (bssA_probe)	95 °C 3 min 95 °C 15 s, 58 °C 60 s 72 °C 3 min	1 50 1	(Beller et al. 2002)
*abcA*	GCGGTGAGGTATTGACCACT (bc_F) TTCGGGCTGACATATCCTTC (bc_R)	95 °C 3 min 95 °C 30 s, 58 °C 30 s, 72 °C 60 s 72 °C 2 min Melt curve from 58 °C to 95 °C with increment 0.5 °C/10 s	1 40 1 1	This study
16S rRNA gene of *Peptococcaceae*	CCTTCGGGTAGACAGGGAGA (PeptoF) AGCCTCTCTAGAGTGCCCAA (PeptoR)	94 °C 3 min 94 °C 30 s, 60 °C 30 s, 72 °C 60 s 72 °C 2 min Melt curve from 60 °C to 95 °C with increment 0.5 °C/10 s	1 35 1 1	This study

^a^Primer names may not correspond to original publication

^b^Y = C or T; S = C or G; V = A or G or C; B = G or T or C; R = A or G

### Benzene carboxylase gene (*abcA*) qPCR primer design and calibration curve

Amplification of the benzene carboxylase gene was done by designing a specific primer pair for *abcA*, a gene described by Abu Laban et al. (2010). The previously designed primer pair bc_F and bc_R (Table 1) (Koressaar and Remm 2007; Untergasser et al. 2012) was evaluated in silico using the publicly accessible NCBI BLAST search tool (Ye et al. 2012). Reactor DNA material was used as template DNA to obtain an amplicon of 175 base pairs. Amplification was carried out in 40 cycles (30 s at 95 °C, 30 s at 58 °C, and 60 s at 72 °C) with an initial denaturation of 3 min at 95 °C and a final elongation step of 2 min at 72 °C on a CFX96 real-time PCR system (Bio-Rad) using TaKaRa Ex Taq polymerase according to the manufacturer’s protocol (TAKARA BIO INC, Otsu, Japan). The amplicons were separated on a 1% agarose gel and extracted using a QIAquick gel extraction kit (Qiagen, Hilden, Germany) according to the manufacturer’s protocol. Gene fragments were cloned into PCR2.1 TOPO vector using the TOPO cloning kit (Invitrogen, Breda, The Netherlands) according to the manufacturer’s protocol with 100 μg/ml ampicillin and 32 μl of 50 mg/ml X-gal. After growth, cells were pelleted, and plasmid was purified according to the manufacturer’s protocol of the QIAprep miniprep system (Qiagen) and sequenced by Eurofins MWG Operon with M13 rev (−29) primer. The obtained sequence was 99% similar to the *abcA* gene sequence identified previously (Abu Laban et al. 2010). The DNA concentration of the cloned *abcA* gene amplicon was determined on a Nanodrop 1000 spectrophotometer (Isogen, De Meern, The Netherlands) to calculate the concentration of the gene copy numbers based on the molecular weight of double-stranded amplicons (Stothard 2000). The stock solution was diluted to 10^10^ gene copies/microliter in MilliQ. The 8-point standard curve was optimized with a lower detection limit of 11.2 gene copies/microliter sample. Amplification of the *abcA* gene was done in a 25 μl reaction mixture as described for *bamA* genes. The thermal amplification profile is shown in Table 1.

### *Peptococcaceae* 16S rRNA gene qPCR primer design and calibration curve

Amplification of *Peptococcaceae* 16S rRNA genes was done using specific primers that were newly designed as described in da Rocha (da Rocha et al. 2010). The specificity of primer pair peptoF and peptoR (Table 1) was evaluated in silico using the public accessible NCBI BLAST search tool, and the predicted amplicon size was 136 bp (Ye et al. 2012). Reactor DNA material was used as template DNA. The standard curve was optimized using a CFX96 real-time PCR (Bio-Rad) with a lower detection limit of 10.5 gene copies/milliter sample. Amplification of *Peptococcaceae* 16S rRNA genes was done in a 25 μl reaction mixture as described for the total bacterial 16S rRNA genes. The thermal cycling conditions are described in Table 1.

### Calculations

First-order degradation rate constants (*k*) were calculated as described previously according to Eq. 1 (van der Zaan et al. 2012).

1 $$ k=\left(\frac{ \log \left({x}_1\right)- \log \left({x}_2\right)}{t_2-{t}_1}\right)+ D $$

where *t*_2_-*t*_1_ is the time interval in days. *x*_1_ is the benzene concentration at time *t*_1_ and *x*_2_ is the benzene concentration at time *t*_2_. *D* is the dilution rate of the continuous culture per day.

The specific degradation rate (*r*) [mg benzene/mg protein/day] was calculated according to Eq. 2:

2 $$ r=\frac{I_b}{\gamma} $$

where *I*_*b*_ is the supply of benzene into the liquid phase of the continuous culture in mg benzene/day and *γ* is the protein content in the culture vessel in milligrams.

The benzene reservoir concentration in the continuous culture vessel (*C*_*r*_) [μM] was calculated as follows (Eq. 3):

3 $$ {C}_r=\frac{C_b^I}{\frac{F_{m+}^I{F}_b^I}{F_b^I}} $$

where *C*_*b*_^*I*^ is the benzene inflow concentration from a 22 mM stock solution and ${F}_m^I$ and ${F}_b^I$ the flow rates of medium and the benzene stock solution in milliters/day, respectively.

The liquid dilution rate of the system (*F*) was calculated according to Eq. 4:

4 $$ F=\frac{F_{m+}^I{F}_b^I}{V} $$

where *V* is the total liquid volume in the culture vessel [L].

## Results

The biofilm culture continuously degraded benzene under nitrate-reducing conditions. At the beginning of biofilm formation at clear hydrophilic surfaces, biofilm was white after which it became brown over time (Fig. S1).

### Continuous culture

#### Benzene degradation and kinetics

**Fig. 2:**
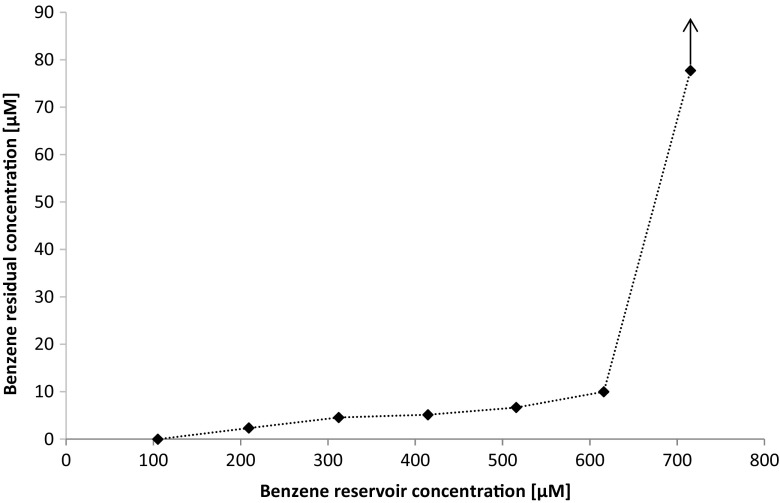
Residual benzene concentrations in the continuous biofilm culture after stepwise increased benzene reservoir concentrations at a liquid dilution rate of 1/day. At a reservoir concentration of 615 μM the benzene concentration continuously increased (indicated by the *arrow* at 715 μM) due to benzene toxicity on the microbial community

Benzene was stably degraded at a reservoir concentration (*C*_*r*_) of 105 μM at four dilution rates from 0.25-2/day. Up to dilution rates of 1/day, benzene was degraded to below the detection limit of the GC method (0.1 μM) with first-order benzene degradation constants increasing from 0.46–1.31/day (Table 2). When the dilution rate was increased to 2/day, the first-order benzene degradation constant was 3.04/day, corresponding to a half-life of 5.5 h (Table 2). However, the residual benzene concentration increased to 1.3 μM. Hence, the dilution rate of 1/day was used to assess the benzene degradation capacity of the continuous culture by stepwise increasing the reservoir benzene concentration from 105 to 715 μM after equilibrium was reached between the liquid and headspace in the culture vessel (Fig. 2). Between benzene inflow concentrations of 105 μM up to 615 μM, benzene was degraded, albeit with steadily increasing residual benzene concentrations proportional to the increase in inflow concentrations. At a benzene reservoir concentration above 615 μM, a threshold value was reached after which the microbial community stopped degrading benzene. The experiment was stopped at a residual concentration of about 70 μM because of the toxic effect of the accumulating benzene on the microbial community.

**Table 2:** First-order benzene degradation rate constants (*k*) and half-lives (hours) at different liquid dilution rates of the continuous culture

Dilution rate [day^−1^]	*k* [day^−1^]	Half-life [hours]
0.25	0.46	36.2
0.50	0.62	26.8
1	1.31	12.7
2	3.04	5.5

At a dilution rate of 2/day, the total protein content in the liquid (2 l) and the white (642 cm^2^) and brown (213 cm^2^) biofilms in the culture vessel were 15, 233, and 2485 mg, respectively, indicating that 99.5% of the microbial biomass in the culture vessel was present in the biofilms. The microbial biofilm and liquid community degraded benzene at a maximal rate of 0.15 μmol benzene/mg protein/day. To determine the possible diffusion limitation of benzene degradation, the stirring rate was varied between 100 and 200 rpm in the culture vessel at benzene reservoir concentrations of 616, 1385, 1478, and 1570 μM and a dilution rate of 0.25/day. During this period, the residual benzene concentration remained below 2.5 μM and did not increase at lower stirring rates of 100 rpm.

At a dilution rate of 0.25/day, benzoic acid was detected at a concentration of 0.18 μM in the liquid phase of the reactor, whereas the concentration of 4-hydroxy benzoic acid and phenol were below the detection limit of the LC-qTOF-MS (<0.18 μM).

After growth of the culture for 20 volume exchanges on a medium without vitamins and ammonium, the community still degraded 100 μM benzene to below the detection limit (1 μM).

Transfers of aggregates of biofilm from the continuous culture grew readily in batch cultures supplied with 10 mM benzoate and 40 mM nitrate. Microcosms inoculated with aggregates of biofilm and addition of 100 μM benzene and 3.6 mM nitrate degraded benzene at an average rate of 1.25 ± 0.66 μM benzene/day with a degradation rate constant of 0.06/day that was about 50 times lower than that obtained in the continuous culture vessel at dilution rate 2/day. The benzene degradation rate was about 700-fold lower than the maximal rate obtained in the continuous culture vessel at dilution rate 2/day. Each time when benzene was depleted in the microcosm, benzene was replenished (Fig. S2).

#### Microbial community

The biofilm and MiSeq sequencing characteristics for the different samples are described in Table 3. The phylogenetic diversity (PD whole tree) was similar for all samples with an average of 4.0 ± 0.2. The number of observed OTUs was higher for the liquid with white biofilm with an average of 139 ± 3 compared with an average of 109 ± 1 for the brown biofilm (Table 3).

**Table 3:** Biofilm and MiSeq sample characteristics

Sample name	Scraped off biofilm area [cm^2^]		Bacterial 16S rRNA genes/cm^2^ biofilm^b^	Sequence reads per sample	OTUs	Phylogenetic diversity
	White biofilm	Brown biofilm				
Sample_1^a^	6.3	0	4.8 ± 2.9 × 10^8^	43,410 ± 33,216	140 ± 3.2	3.9 ± 0.2
Sample_2	6.3	0	4.2 ± 2.0 × 10^8^	52,014	138	4.2
Sample_3	22.3	4	9.8 ± 4.3 × 10^9^	42,427	108	3.8
Sample_4	22.3	8	13 ± 5.7 × 10^9^	53,124	109	4.1

^a^Average of three samples

^b^Calculated based on liquid and biofilm protein (white biofilm 233 mg, brown biofilm 2485 mg) in the sample

**Fig. 3:**
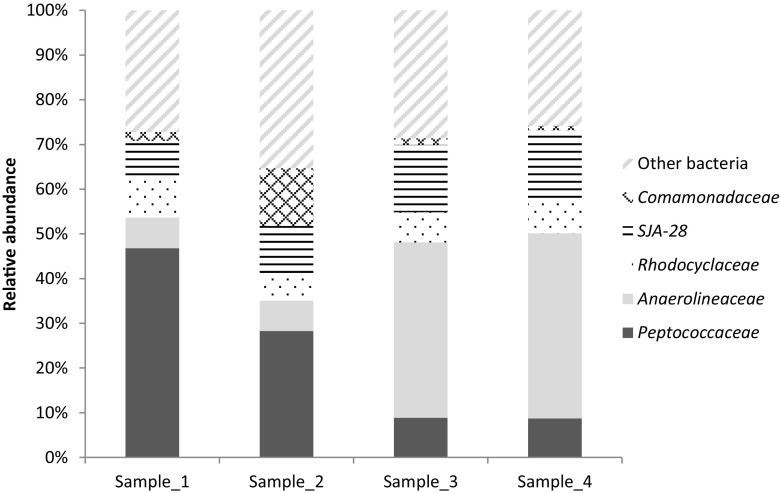
The relative abundances of partial bacterial 16S rRNA genes at family level in the continuous culture. Samples 1 and 2 contained 11% biofilm and 89% culture liquid protein. Sample_3 contained 70% biofilm and 30% liquid protein biomass. Sample_4 contained 100% biofilm. Only families with a relative abundance of 10% or higher in at least one of the biofilm samples are shown. Families occurring at lower relative abundances are shown together as “other bacteria”

Based on the MiSeq community analysis data, the relative abundance of *Peptococcaceae* in the samples containing brown biofilm was 8.8 ± 0.0% and ranged from 28 to 49% in the liquid with white biofilm (Fig. 3). MiSeq analysis also indicated that members of the *Anaerolineaceae* were relatively predominant in the two samples containing brown biofilm with 39 and 41% relative abundance in samples 3 and 4, respectively (Fig. 3). Other relatively predominant families in both the liquid with white biofilm and brown biofilm were *Rhodocyclaceae* (8.1 ± 1.5%), SJA-28 (10.8 ± 3.6%), and *Comamonadaceae* (3.5 ± 4.6%).

Based on qPCR analysis, sample 4 contained 16 ± 4.1 × 10^7^ *Peptococcaceae* 16S rRNA genes/cm biofilm. The relative abundance of *Peptococcaceae* calculated using qPCR was similar to that obtained by MiSeq analyses (Fig. S3).

**Fig. 4:**
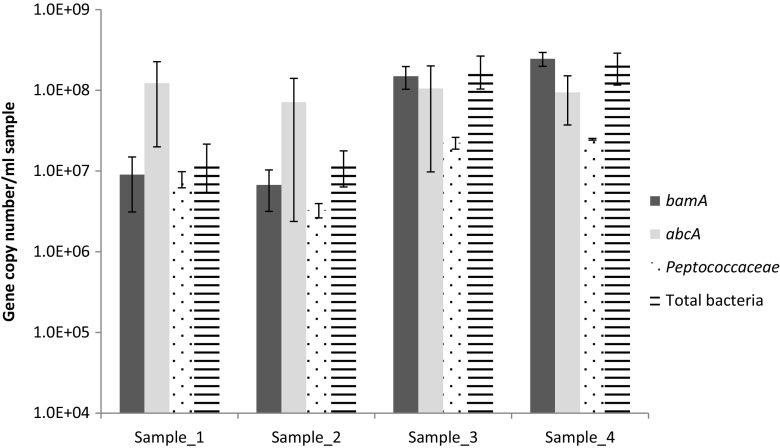
qPCR analyses of *bamA*, *abcA*, and 16S rRNA genes of *Peptococcaceae* and total bacteria. Samples 1 and 2 contained 11% white biofilm corresponding to 4.8 ± 2.9 × 10^8^ and 4.2 ± 2.0 × 10^8^ bacterial 16S rRNA genes/cm^2^ biofilm, respectively. Sample_3 contained 70% biofilm corresponding to 9.8 ± 4.3 × 10^9^ bacterial 16S rRNA genes/cm^2^ biofilm. Sample_4 contained 100% biofilm corresponding to 13 ± 5.7 × 10^9^ bacterial 16S rRNA genes/cm^2^ biofilm. *Error bars* represent standard deviations based on analytical replicates of the same reactor sample

The functional gene *bssA* was not detected in the culture (<3.1 gene copies/ml sample). The other two functional genes studied here, *bamA* and *abcA*, were detected in high copy numbers in all samples, i.e., 10 ± 2.6 × 10^7^ gene copies/ml sample and 9.9 ± 8.1 × 10^7^ gene copies/ml sample, respectively (Fig. 4). *Peptococcaceae* 16S rRNA gene copy numbers were generally about tenfold lower in the different samples than those of the *abcA* gene.

### Continuous culture with retentostat

**Fig. 5:**
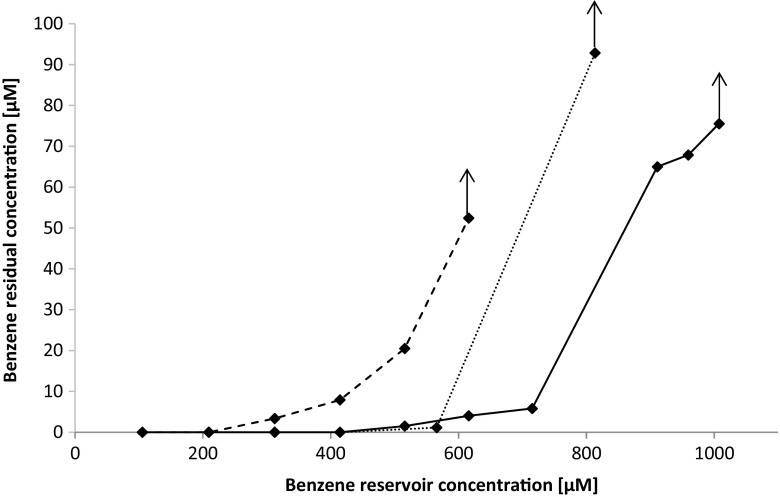
Benzene residual concentrations in the continuous culture with retentostat finger. The *black line* indicates the first (initial) experiment, the *dotted line* the second experiment at 88 days, and the *dashed line* the third experiment at 118 days after the initial experiment, respectively. The arrows show reservoir concentrations of 1007, 840, and 616 μM, for the first, second, and third experiments, respectively, where benzene degradation was continuously increased

In an attempt to further increase the activity of the benzene-degrading culture, a recirculation system with retentostat finger was added to retain biomass. Reservoir benzene concentrations were increased stepwise from 105 to 1000 μM to obtain the maximum degradation capacity of the culture with the retentostat finger at a dilution rate of 0.7/day. Initially after the addition of the retentostat finger the first-order benzene degradation rate constant was 3.93/day and the reservoir concentration at which the system collapsed and the microbial community was not able to degrade benzene due to toxicity was 715 μM at a dilution rate of 0.7/day (Fig. 5). After two subsequent replications of the retentostat experiment, the benzene-degrading capacity of the culture decreased and the culture stopped degrading benzene at lower benzene reservoir concentrations of 566 and 209 μM, respectively (Fig. 5).

## Discussion

The goals of this study were: (i) to determine the maximum anaerobic benzene degradation capacity of a continuous biofilm culture, (ii) to reveal the initial step in benzene degradation, (iii) to characterize the microbial composition of the liquid and biofilm-associated biomass at different ages using high-throughput sequencing of PCR-amplified 16S rRNA gene fragments, and (iv) to obtain a highly active benzene-degrading community. The benzene degradation rate constant (*k*) of 3.04/day obtained in this study in the continuous culture vessel is about four times higher than the previously observed 0.70 ± 0.12/day that was obtained using the same microbial culture under denitrifying conditions (van der Zaan et al. 2012). To our knowledge, this study represents the highest anaerobic benzene degradation rate constant reported under denitrifying conditions (Table 4).

**Table 4:** First-order anaerobic benzene degradation rates under denitrifying conditions

Enrichment culture source	Benzene degradation rate constant [day^−1^]	References
Benzene-degrading continuous biofilm culture in reactor from a benzene polluted industrial location in the northern parts of the Netherlands	3.04	This study
Benzene-degrading continuous biofilm culture from a benzene polluted industrial location in the northern parts of the Netherlands	0.7 ± 0.12	(van der Zaan et al. 2012)
Batch enrichment culture from an oil refinery in Oklahoma	0.29^a^	(Ulrich et al. 2005)
Benzene-degrading enrichment culture in microcosms from an oil refinery in Oklahoma	0.21^a^	(Ulrich and Edwards 2003)
Batch enrichment culture from a coal-tar contaminated site in Glens Falls	0.15^a^	(Liou et al. 2008)
Pure culture of *Bacillus cereus* from a gasoline contaminated soil	0.08^a^	(Dou et al. 2010)
Enrichment from gasoline contaminated groundwater in microcosms	0.05^a^	(Kasai et al. 2006)

^a^Calculated based on the benzene degradation in the discussed studies

Besides obtaining the benzene degradation rate constant in this study, a specific benzene degradation rate was calculated based on the amount of proteins in the reactor vessel. In previous studies aerobic degradation rates of 10 to 321 μmol benzene/mg dry weight/day were reported (Suarez and Rifai 1999). Assuming an average protein content of 55% (protein content of *E. coli*) of dry mass for bacteria (Milo 2013), these rates correspond to 5.5 to 176.5 μmol benzene/mg protein/day. The anaerobic benzene degradation rate of 0.15 μmol benzene/mg protein/day found in the present study is therefore 37 to 1177-fold lower than previously described aerobic degradation rates. In a previous study, an anaerobic degradation rate of 4.9 to 9.3 μmol benzene/mg protein/day under denitrifying conditions was determined using enrichment cultures in microcosms from a gas station and a swamp in Canada (Ulrich and Edwards 2003). These rates are 33 to 61-fold higher than the anaerobic benzene degradation rate found in the present study, which could be explained by the fact that more benzene degraders per protein were present and fewer organisms growing on other substrates like ammonium or vitamins supplied in our medium with concentrations of 68.2 and 2.6 mg/l, respectively. According to the growth yield of anammox bacteria (0.066 mol biomass carbon/mol ammonium) a maximum of 250 μM carbon biomass could be produced with the supplied ammonium (Strous et al. 1998). This corresponds to 42% of the carbon supplied as benzene. The amount of carbon supplied in vitamins corresponds to 124 μM, assuming an average molecular vitamin formula of C_12_H_16_N_2.6_O_3.4_. This corresponds to 21% of the benzene carbon supplied. The bacterial community is able to degrade the benzene without the vitamins and ammonium in the medium, indicating that the supply of vitamins and ammonium in the medium is not required for benzene degradation. A higher amount of benzene degraders per protein in previous studies could be due to the fact that the microcosms were enriched through several serial transfers in the study of Ulrich et al. (Ulrich and Edwards 2003). In addition, other explanations that could support this higher rate are: (i) faster benzene-degrading organisms, (ii) enrichment using a batch study corresponding to a high maximal growth, and benzene degradation rate compared with limited substrate availability in a continuous culture used in this study corresponding to a lower maximal growth and benzene degradation rate. The rate of 0.15 μmol benzene/mg protein/day found in the present study at a dilution rate of 2/day may not be the maximal degradation rate for the residing microbial community, since this rate was measured under benzene limiting conditions. Experimental data indicated that benzene degradation was not limited through benzene diffusion from the bulk liquid into the biofilm (Nielsen 1987; Rittmann and McCarty 1980). This suggests that the maximal benzene degradation capacity obtained in this study was limited by the degradation kinetics of the microorganisms and/or the available biofilm surface in the culture. An increased benzene-degrading capacity of the bioreactor is expected if more surface area for biofilm formation would be available. The benzene degradation rate decreased in this study when a liquid inoculum with biofilm aggregates from the reactor was used in a microcosm. The degradation rate in the microcosm was lower than that observed in the continuous culture since solely liquid with biofilm aggregates was used as inoculum instead of pure biofilm culture. Our results show that most of the benzene degraders reside in the biofilm since 99.5% of the microbial biomass was present in the biofilms as was suggested before (van der Zaan et al. 2012).

The presence of benzoic acid (0.18 μM) in the culture vessel, and absence of phenol suggests that benzene is degraded via an initial carboxylation (Luo et al. 2014). Other observations that support this are (i) the presence of the benzene carboxylase encoding *abcA* gene, and (ii) the absence of *bssA* functional genes as determined by qPCR. The higher counts of the *abcA* gene compared with *Peptococcaceae* 16S rRNA gene copies may indicate that there are multiple *abcA* gene copies in the DNA of *Peptococcaceae*, that the primers are targeting similar genes, or that the *abcA* gene is also present in other organisms. While being beyond the scope of the current study, future experiments should include analysis of the putative benzene carboxylase gene at the RNA level to confirm that the *abcA* gene is expressed during growth on benzene.

A similar phylogenetic diversity was noted for all the liquid and biofilm samples, but lower numbers of OTUs in the brown biofilm indicate that the phylotypes observed in the brown biofilm were phylogenetically more distantly related than those present in the liquid phase and the white biofilm, as the diversity metric used here, PD whole tree, takes phylogenetic distance of community members into account (Faith 2006). The fact that benzoate degraders unable to degrade benzene were present may contribute to the high bacterial diversity within the biofilm reactor. A rapid and extensive growth was indeed observed in transfers of the continuous culture in microcosms with benzoate.

Relatively high numbers of *Anaerolineaceae* 16S rRNA genes were detected in the biofilms. Currently, a few members of the family *Anaerolineaceae* are described and isolated that are strict anaerobes able to grow on oil components and often found under sulfate-reducing or methanogenic conditions (Liang et al. 2015; Sutton et al. 2013; Yamada et al. 2006). *Anaerolineaceae* family members grow by fermentation, generating fermentation intermediates such as acetate and hydrogen from alkanes, which can be oxidized by other community members (Liang et al. 2015; Yamada et al. 2006). Further, compiling evidence points towards potential roles of *Anaerolineaceae* in the degradation of aromatic compounds. For example, uncultured *Anaerolinea* spp. have been shown to scavenge organic compounds from lysed cells (Sekiguchi et al. 2003). Microbial community analysis of an anaerobic sequencing batch reactor (ASBR) revealed members of the family *Anaerolineaceae* as putative anaerobic phenol degraders (Rosenkranz et al. 2013). Kümmel et al. (2015) also obtained enrichments growing on polycyclic aromatic hydrocarbons (PAHs), benzene, toluene, ethylbenzene, and xylene (BTEX), highly enriched in *Anaerolineaceae.* It is possible that *Anaerolineaceae* are primary benzene degraders in the biofilm or that they are enriched within the biofilm because they oxidize benzoate excreted by primary benzene carboxylating *Peptococcaceae* (van der Zaan et al. 2012). Less than 0.5% of the benzene supplied to the reactor was detected as benzoate. *Peptococcaceae* may excrete benzoate from leaky cells or benzoate might be produced at the membrane as suggested for *Dechloromonas* strain RCB (Chakraborty and Coates 2005). Therefore, it is reasonable to assume that *Peptococcaceae* consume a major fraction of the benzene. It has previously been suggested that *Peptococcaceae* may be responsible for the first step in benzene degradation and *Azoarcus* scavenges electrons from benzoate, produced by leaky of lysed cells (Luo et al. 2014).

Being another predominant family observed in our study, members of the *Comamonadaceae* have been shown to be able to degrade phenol and toluene under denitrifying conditions (Arai et al. 1998; Sun and Cupples 2012). Recently, a *Comamonas* sp. was isolated from our reactor, which was not able to grow on benzene but grew on phenol, p-hydroxybenzoate, and methyl-hydroxybenzoate with nitrate (Balk, personal communication). Possibly, also other metabolites such as phenol are produced in the continuous culture. Members of the family SJA-28 are affiliated to the phylum *Ignavibacteria*. Current knowledge about *Ignavibacteriaceae* is limited. Kümmel et al. (2015) observed an increase in *Ignavibacteriaceae* in an enrichment culture obtained from a sediment sample contaminated with PAHs and BTEX. Besides SJA-28, *Rhodocyclaceae* were also relatively predominant. It was previously suggested that *Rhodocyclaceae* are involved in nitrate reduction and syntrophically associated with the anaerobic benzene-degrading *Peptococcaceae* (Luo et al. 2014; van der Zaan et al. 2012). The recently isolated *Pseudomonas stutzeri* strain BN and a bacterium belonging to the *Bacillus* sp. from our reactor were not detected in the samples based on community analysis. It is possible that these strains are minor degrading populations, but based on the low relative abundance not responsible for the large amount of benzene degradation. The recently isolated *Ochrobactrum* sp. was present in our community data.

After the addition of the retentostat finger to further increase the activity of the benzene-degrading community, the degradation rate constant was initially higher than without retentostat. However, replication of the experiment with the retentostat finger resulted in a decreased benzene degradation capacity. This decreased degradation capacity may be explained by the selection of microbes with a high affinity and low maximum consumption rate for benzene. During retention of biomass more microbial cells in the culture vessel are expected to share the same amount of substrate. This induces starvation, and physiological adaptation of the benzene-degrading microorganisms or adaptation of the community composition to low substrate concentrations, generally resulting in a reduced maximum degradation rate (Ercan et al. 2015; Kjelleberg et al. 1993; Schut et al. 1993). At the same time a selective pressure is imposed, favoring the enrichment of microorganisms with high affinity for benzene, which may be expected to have relatively low maximal growth and degradation rates (Gottschal 1993). When the continuous culture was restored after the retentostat experiments, it degraded benzene to below 0.1 μM up to dilution rates of 4/day at a benzene reservoir concentration of 105 μM. This indicates that the diverse microbial community in the biofilm readily adjusts to changing conditions and was able to restore its function after a system perturbation, indicating resilience at least across the varying conditions applied in this study.

This study suggests that other bacteria besides *Peptococcaceae* have an important role in the anaerobic benzene degradation. Our data suggest that benzene is degraded through an initial carboxylation. This study also represents the highest anaerobic benzene degradation rate constant published so far and confirms that the microbial community in biofilms can be important for benzene degradation under conditions of continuous supply. It is therefore important to further study the significance of biofilms during natural attenuation at contaminated sites. This may also broaden our options to use biofilms for stimulated bioremediation through biostimulation and bioaugmentation at contaminated groundwater systems.

## Electronic supplementary material

ESM 1 (PDF 206 kb)
