# Supplementary material for: Benzene degradation in a denitrifying biofilm reactor: activity and microbial community composition
Source: Appl Microbiol Biotechnol. 2017 Mar 20;101(12):5175–88. doi: 10.1007/s00253-017-8214-8 (PMC5486827; doi:10.1007/s00253-017-8214-8)
Supplement: Supplementary file 1 — (PDF 206 kb) [file 253_2017_8214_MOESM1_ESM.pdf]

Applied Microbiology and Biotechnology

Benzene degradation in a denitrifying biofilm reactor: activity and microbial community composition

Marcelle J. van der Waals[#1,2], Siavash Atashgahi[2], Ulisses Nunes da Rocha[3], Bas M. van der Zaan[1], Hauke Smidt[2] and Jan Gerritse[1]

[1] Deltares, Subsurface and Groundwater Systems, Princetonlaan 6, 3584 CB Utrecht, the Netherlands

[2] Wageningen University & Research, Laboratory of Microbiology, Stippeneng 4, 6708 WE Wageningen, the Netherlands

[3] VU University of Amsterdam, Department of Molecular Cell Physiology, De Boelelaan 1085, 1081 HV Amsterdam, the Netherlands

# Corresponding author

Phone +31 646914584, Fax +31 88 3357856, E-mail: Marcelle.vanderwaals@deltares.nl

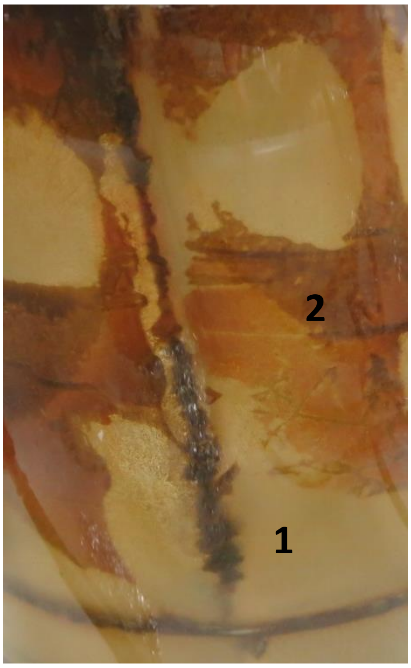

Figure S1 White (1) and brown (2) biofilm attached at the glass wall and metal surfaces in the continuous culture

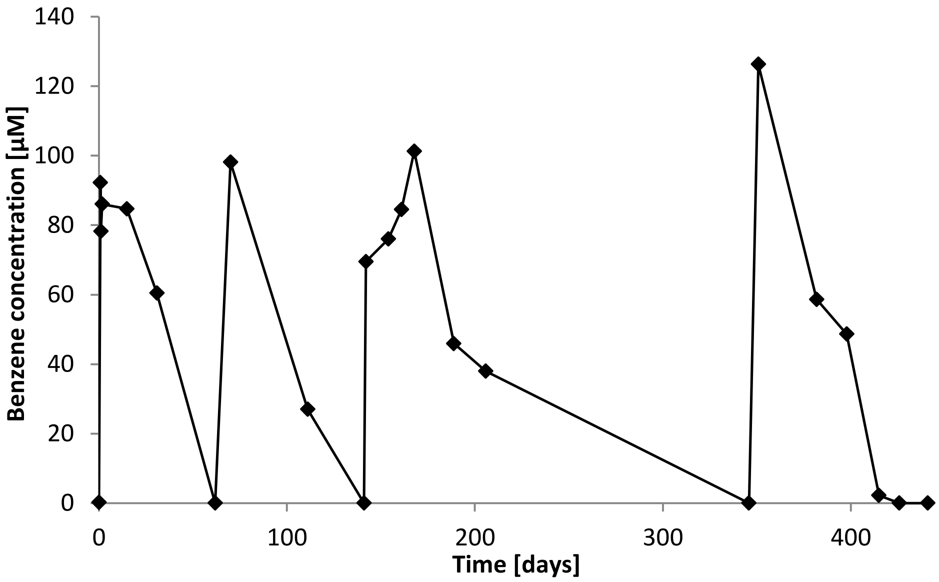

Figure S2 Time course analysis of benzene concentration in the microcosm with transferred liquid from the continuous culture supplied with 100 µM benzene and 3.6 mM nitrate. After each benzene depletion, benzene was replenished

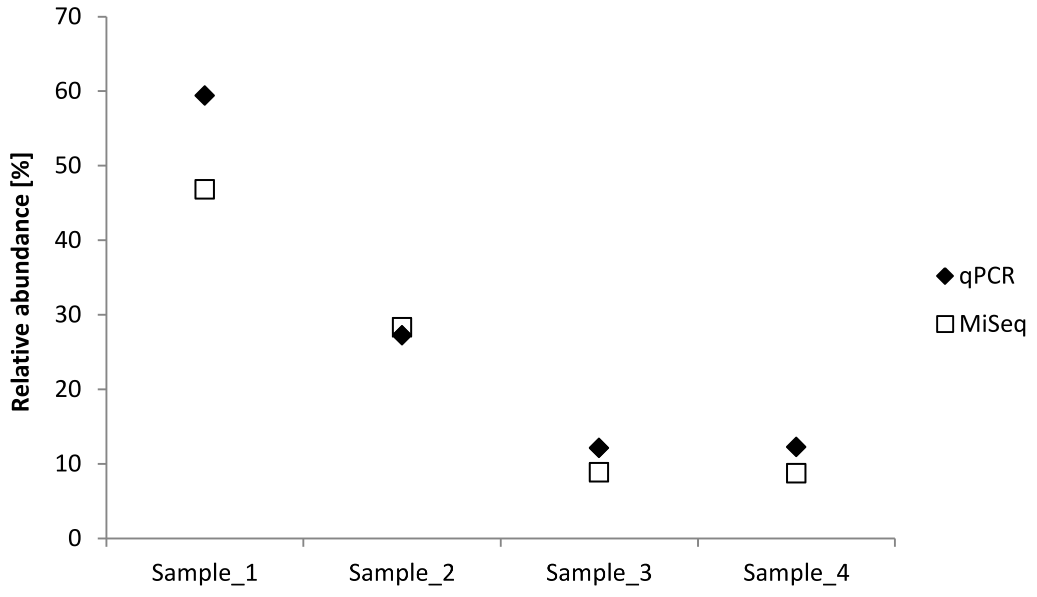

Figure S3 Relative abundance of *Peptococcaceae* versus total bacterial 16S rRNA gene copies in the different samples, calculated using qPCR (closed diamonds) and MiSeq (open squares)
